# The Advantage of the 5G Network for Enhancing the Internet of Things and the Evolution of the 6G Network

**DOI:** 10.3390/s24082455

**Published:** 2024-04-11

**Authors:** Georgios Gkagkas, Dimitrios J. Vergados, Angelos Michalas, Michael Dossis

**Affiliations:** 1 Department of Computer Science, University of Western Macedonia, 52100 Kastoria, Greece; g.gkagkas@uowm.gr (G.G.); mdossis@uowm.gr (M.D.); 2 Department of Electrical and Computer Engineering, University of Western Macedonia, 50100 Kozani, Greece; amichalas@uowm.gr

**Keywords:** IoT (Internet of Things), 5G networks, MTC (machine-type communication), D2D (device-to-device), wireless communication

## Abstract

The Internet of Things (IoT) is what we have as a great breakthrough in the 5G network. Although the 5G network can support several Internet of Everything (IoE) services, 6G is the network to fully support that. This paper is a survey research presenting the 5G and IoT technology and the challenges coming, with the 6G network being the new alternative network coming to solve these issues and limitations we are facing with 5G. A reference to the Control Plane and User Plane Separation (CUPS) is made with IPv4 and IPv6, addressing which is the foundation of the network slicing for the 5G core network. In comparison to other related papers, we provide in-depth information on how the IoT is going to affect our lives and how this technology is handled as the IoE in the 6G network. Finally, a full reference is made to the 6G network, with its challenges compared to the 5G network.

## 1. Introduction

The 5G network is using technologies that are not used in 3G, 4G, and LTE. Such technologies are Low Power Wide Area (LPWA) and long-distance communication. IoT is the technology that allows devices to use the 5G network and then interconnect a series of applications. The study in reference [1] provides a detailed analysis and discussion regarding the use of LPWA in the wireless network concerning coverage, power efficiency, and Quality of Service (QoS).

Reviewing the study of evolution from 1G to 6G and their network speed references [2] presents a comprehensive study where we can easily identify all their characteristics. The 5G and 6G networks are the technologies used for the IoT connectivity of devices, including challenges such as indoor and outdoor penetration. The New Radio (NR) is quite an important part in the IoT implementation, which includes the use of multiple input, multiple output (MIMO) antennas as part of the signal transmission, as well as the use of IPv6 for backhaul connectivity and device-to-device (D2D) communication.

With the term 5G meaning 5th-generation technology and IoT meaning the Internet of Things, both technologies are used to connect people and devices with the use of applications and sensors. Smartphones and IoT devices have changed the way people interact with each other. The 5G and 6G networks allow for the possibility to use extremely high volumes of data and connect multiple devices at the same time. Applications such as social media, online gaming, and high-definition (HD) videos can use 5G and 6G networks, leading to new economic and business standards. Cloud services will allow information sharing and enable collaboration between industries.

The machine-to-machine (M2M) communication is analyzed in reference [3], which is also a part of this type of communication. This includes communication and control of vehicles, which require an immediate response, remote surgery, or automatic braking in cars, as well as meteorological meter reporting in real time. M2M communication plays an important role in the 5G network. The 5G networks will enrich cellular M2M communication, reducing end-to-end (E2E) delay, increasing battery operational time, and allowing huge number of devices to be connected per cell.

In the 5G network, Open Radio Access Network (O-RAN) is a solution to deploy the Radio Access Network (RAN) at a low cost since the deployment of the 5G network requires a high cost. With O-RAN, the mobile operators have more flexibility compared to traditional RAN deployment, applying virtualization and intelligence. With the use of O-RAN, which is based on open interfaces, mobile operators will bring the cloud and interoperability concept to RAN, ensuring intelligence and optimization of the infrastructure.

The study of CUPS [4], which is a foundation for the “Network Slicing”, in the 5G core network allows for the possibility of independent scalability. The key advantages of independent geographical location and unlimited scalability of the control plane, as well as the user plane, are the main characteristics of CUPS. There can be many user planes (UPs) deployed where there can only be a few Policy and Charging Rule Functions (PCRFs) and Online Charging System (OCS) associations. Software Defined Networks (SDNs) and Virtual Network Functions (VNFs) are the ones that enable a deep data analysis, providing all the needed monitoring and measurement reports of the networks and the applications.

The 5G and 6G radio network functions are in higher frequencies as a result of fewer penetration capabilities but possess higher spectrum utilization, which can be used to increase the capacity of the cells without increasing the number of sites. The energy consumption is independent of the network load, which means that even at low loads, the energy consumption is relatively high. The 6G network will be able to provide terahertz communication and improvement in coverage and data rate. The 6G network will provide a new communication experience with enhanced conventional mobile communication, accurate indoor positioning, high-quality communication services, and even holographic communications. The 6G network is expected to provide video in-person meetings that support a realistic projection of real-time three-dimensional (3D) images, along with voice as mentioned in reference [5], which is an extremely large bandwidth requirement. Human-bond communication is another concept that will be supported with the use of the 6G network. With human-bond communication, we will be able to share and access the five basic human senses [2]. As a result, 6G will be able to collect biological information such as diseases and emotional detection.

Some introductory related work can be found today in references [1,2], which are holistic survey papers on the opportunities and role of 6G within IoT technology and its applications. Reference [3] is a detailed presentation of IoT data-network fundamentals exploring applications such as smart transport, smart-health, smart city, and smart industry. Reference [4] is a comparative discussion highlighting the key challenges of future standardization in IoT, and reference [5] is an experiment based on the adoption of three-dimensional (3D) holographic displays in applications such as smart glasses, car heads-up displays, etc.

**Figure 1:**
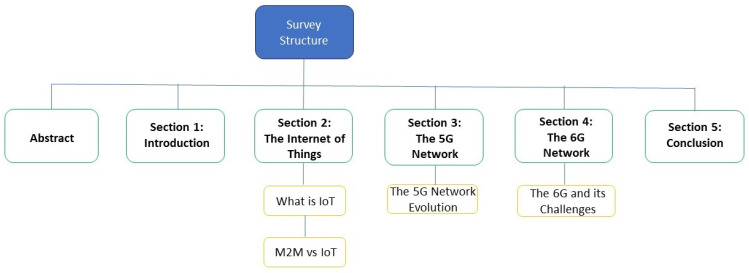
Survey structure.

This paper is organized as follows (Figure 1): In Section 2, the technologies of IoT and M2M communication are explored discussing factors that we need to consider, such as coverage, scalability, diversity, battery life, and device cost, as well as the needed encrypted protocols that are used for data transmission. In Section 3, we discuss 5G mobile network technology and all of its advantages, such as high data-communication rates, low latency, reliability, and availability. CUPS functionality is introduced as a part of network slicing, allowing for the possibility of providing independent scalability of the control and the user plane based on the network needs and requirements. Geographical redundancy is also part of the above. In terms of security IPsec, ensures secure communication. In Section 4, we see that 6G will be the network to interconnect all things. The 6G network will actually be the connection of intelligence with great capacity and latency improvement compared to what we currently have in 5G, with services such as surreal virtual reality, medical imaging, car self-driving, smart home, holographic and precision communications, and much more. Finally, in Section 5, we conclude the article.

The list of abbreviations used in the paper can be found at the end of the paper.

## 2. The Internet of Things

### 2.1. What Is IoT

**Figure 2:**
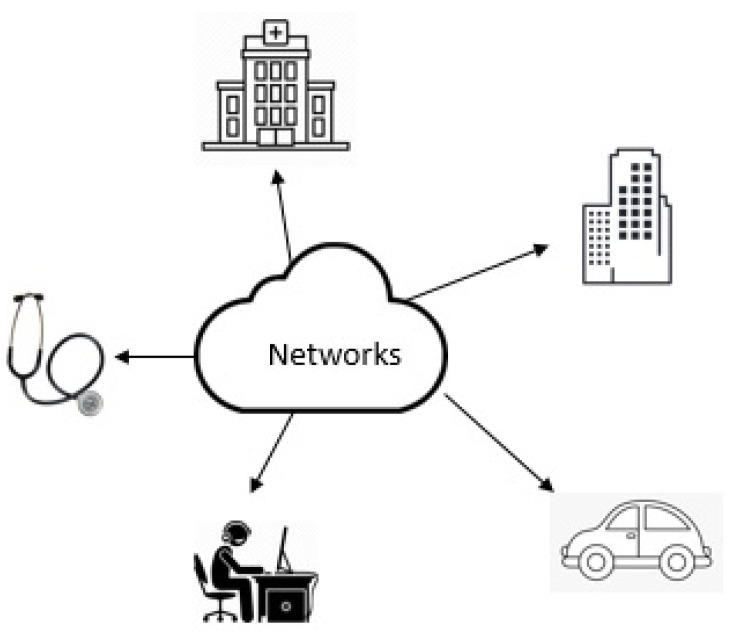
The IoT devices.

The IoT will change businesses and services. Based on our study, when we refer to “Things,” this represents the physical layer of the Open Systems Interconnection (OSI) model. All the physical devices can communicate with the network infrastructure (Figure 2). A physical thing can be mapped to a virtual thing within the information world, although a virtual thing can exist by itself in the information world.

**Figure 3:**
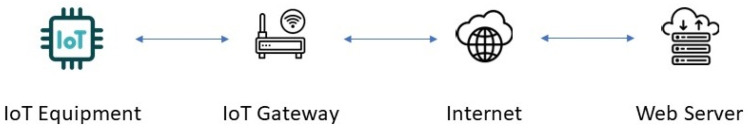
The IoT architecture.

The architecture of the IoT device connectivity can be seen in Figure 3. It consists of the IoT physical devices, which constantly send information from the sensors attached to them, the IoT gateway which aggregates all the data received from the IoT devices, the internet connectivity which allows the communication with the web server, and the web server who is responsible for hosting all of the applications allowing the data exchange between all the connected devices.

Based on our research on the IoT technology, we see that IoT devices are equipped with communication and data-capture capabilities, as well as a series of sensors and data-storage and data-processing capabilities. The IoT devices collect and provide information to the communication network for further processing. So, when we refer to devices, those devices can communicate with each other in different ways: directly, locally through a gateway, and over the communication network.

**Figure 4:**
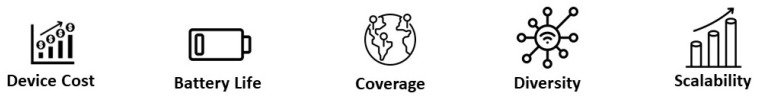
The IoT challenges.

The communication network transfers all of the captured data from the IoT devices to the applications and other devices as instructions from the applications to the devices. There are IoT devices that need a two-way communication (uplink—downlink) in order to monitor and control the IoT devices. This is quite a hot topic in IoT technology since we need to take into consideration factors such as coverage, scalability, diversity, battery life, and, of course, the cost of devices (Figure 4).

The use of O-RAN in IoT will provide flexibility in terms of connectivity for the series of applications, such as e-health, security, and medical care, overcoming any possible restrictions in terms of IoT connectivity. O-RAN can support massive IoT device connectivity as it requires low battery consumption and provides large coverage with low throughput. O-RAN architecture adds additional interfaces and controllers in order to meet all of the IoT service requirements. The greatest feature of O-RAN is its RAN intelligent controllers (RICs), which allow for the possibility of providing better performance than the legacy RAN networks and using Artificial Intelligence- (AI) and machine-learning-based (ML) algorithms, optimizing the network in terms of load balancing and slicing control while enabling multiple tenants and handover management. As mentioned above, all of the components of O-RAN can be virtualized using Kubernetes platforms, offering a dynamic adjustment of all of the resources. That is based on what the network really needs, such as reduced power consumption.

Battery life is one of the most important factors in IoT technology. Latest research has shown that IoT devices battery lifetime is about 10 years’ time at the most. Most of the IoT devices have limited energy resources, so low power consumption for both data transmission and self-protection is needed. A duration time of more than 10 years of lifetime can be achieved by introducing the power-saving mode (PSM) as studied in references [6,7], and in the extended discontinuous reception functionality (eDRX). These features allow the IoT devices to contact the network on a peer-need basis, meaning that it can stay in sleep mode for minutes, hours, or even days.

The current consumption of an IoT device should be lower than 1 μA. One good example of that would be a smart wearable device, such as a smart watch or smart clothes with built-in sensors, that transmits and receives very small amounts of data, such as body temperature or heartbeat measurements, towards or from the network periodically, including large time frames of transmission inactivity. This device, constantly records measurements from the human body, processes and calculates all of those measurements internally, consumes very small amounts of energy and periodically transmits updates to the network. The only disadvantage on that will be that the device cannot respond to possible requests during the idle connectivity timeframe. A good solution to the above, referring to the reduction of battery dependency, is the use of an energy-harvesting system. Such a system could be a solar-power collector, a wind-energy collector, or even a human-body-movement-energy collector. For example, a sleeping person could produce about 80 watts of energy, or a short-distance walking person could produce about 160 watts. All of this energy could be stored in batteries for the immediate or future use of a low-power, wearable IoT device.

Today’s existing IoT work is presented in the following papers: Reference [6] is a paper presenting the Institute of Electrical and Electronics Engineering (IEEE, Piscataway, NJ, USA) 802.11 Wireless Local Area Network (LAN) architecture for IoT systems. In reference [7], the Extended Discontinuous Reception (eDRX) protocol for energy saving on the smart grid is presented. The security implications of such systems are examined in reference [8], which focuses on the Advanced Encryption Standard (AES) used for communication security within wireless sensor networks. In addition, reference [9] presents a comparison of encryption and decryption times of the AES common nodes, and reference [10] focuses on the lightweight algorithms of the IoT solutions.

The study and analysis of the encryption algorithm chosen, which is presented in references [8,9], is one of the most important factors to consider for the calculation of the power lifetime of an IoT device. The more secure the algorithm used, the more energy-consuming it is. Such protocols are the AES using 128-bit size blocks and the Elliptic Curve Cryptography (ECC), which requires a smaller key size as a result of its fast processing and smaller memory requirements. Piccolo is another protocol studied in reference [10] that is used in IoT devices, which seems to be the most energy-efficient method than others providing the maximum lifetime with the lowest consumption.

IoT applications are possible to be on a ‘fit and forget’ base. The less frequently the User Equipment (UE) listens for paging, the higher the latency is, and also the longer the lifetime of the battery is. The power-saving mode (PSM) is similar to power off, but with the UE remaining registered to the network and with no need to re-attach or re-establish a Packet Data Network (PDN) connectivity. A UE, using the PSM functionality, is available for mobile-terminating services during its connected mode, which is caused by a mobile-originated event like signaling and data transferring, and for the period of an active time, which is after the connected mode. The PSM is intended to be used by UEs that are expecting only infrequent mobile originating and terminating services and can accept latency in the mobile-terminating communication.

The eDRX, on the other hand, has been studied in reference [7], which improves the power efficiency of mobile devices while they remain reachable through paging, an ideal feature for applications with stricter reachability requirements than PSM. eDRX can be used together with the PSM without any issues. For devices that have the eDRX feature enabled, the Serving General Packet Radio Service (GPRS), together with Support Node Mobility Management Entity (SGSN-MME), supports the delaying of paging requests until the device is in the paging window. During the attach-request procedure, the user equipment (UE) includes the eDRX parameters. If the Mobility Management Entity (MME) accepts the request, the MME includes the eDRX parameters in the attach-accept message-request response. What the eDRX does is it actually extends the sleep cycles in inactive mode with configurable values varying from seconds to minutes. This results to the reduction of the battery power need from the IoT device, and also it reduces any maintenance cost such as a replacement of the battery comparison by just using the PSM. eDRX ensures a certain reachability period of the IoT device providing significantly improved downlink reachability.

Functions for high-latency communication (HLC), as studied in reference [7], may be also used to handle the mobile-terminated (MT) communication, with the UEs being unreachable while the power-saving functions are used. High latency refers to the initial response time before the normal exchange of the packets establishment, which refers to the time it takes before a UE has woken up from its power-saving state and responded to the initial downlink packet. The HLC is used in order to achieve full synchronization between the application server and the UEs using PSM or eDRX, optimizing the signaling between the mobility-management entity (MME) and the serving gateway (SGW) when the payloads are buffered.

The basic areas that can affect the IoT devices’ lifetime are:

- The technical characteristics of the IoT devices, such as the central processing unit (CPU), memory capacity, signal level, operating system, battery capacity, and power consumption of the processor.
- The network topology of the IoT devices system, which determines the amount of information passing through.
- The functional characteristics of the IoT devices, such as data-transfer protocols, security, and encryption methods used.
- The frequency and transmission of the collected data.

Based on all those factors we can calculate IoT device’s lifetime. How often the IoT device records the measurements from its sensor is a basic parameter. For the calculation of the energy consumption during data transmission, we need to consider the following parameters:

- Central processing unit (CPU) frequency
- Transmitted packet size
- Channel bitrate
- Total number of processed algorithm commands
- Sleep mode timeframe
- The channel bit rate

As open research challenges, we do consider series of issues within IoT technology. A major one is the appliance of AI technology in terms of resource handling and services. Quality of Service (QoS) is another one in terms of throughput reliability, availability, reusability, transmit time, and latency. Security requirements are another major topic in order to handle concerns such as privacy authentication and authorization. Security is a sensitive area since issues such as end-to-end communication between gateway sensors and the cloud need to be addressed. Scalability, such as handling mass IoT connectivity and operation and maintenance, are also hot topics.

### 2.2. M2M vs. IoT

**Figure 5:**
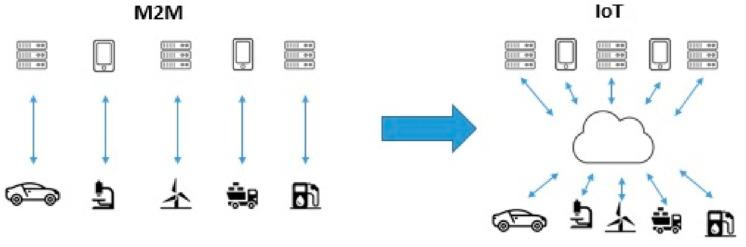
Machine-to-machine (M2M) communication compared to the Internet of Things (IoT) communication.

Existing work on M2M and IoT communication contains several papers that are presented as follows: Ref. [11] is a paper providing case studies of IoT business-based transformation. Ref. [12] is a detailed review of IoT data-network infrastructure that explores applications such as smart healthcare, smart transportation, smart industry, and smart cities. Ref. [13] is a book that explores M2M communication, smart-service applications, the challenges of the world of IoT, and the risks of such connectivity. Ref. [14] covers M2M system architecture, performance-management techniques, services, and standardization. Ref. [15] is a detailed survey of M2M communications focusing on the Long-Term Evolution-Advanced (LTE-A) networks and an overview of the expected 5G M2M services. Ref. [16] is a survey paper of IoT technology, highlighting the key challenges of its future standardization. Ref. [17], which is a survey work on M2M communication with its challenges and congestion control in Third-Generation Partnership Project (3GPP) LTE/LTE-A networks. Ref. [18] is an article describing the architecture of machine-type communication (MTC) within 3GPP systems, including overload protection mechanisms and the functionality of new network elements. Ref. [19] is a paper related to M2M communications linking the Internet cyber world and physical systems. Reference [20] is a comprehensive review of the M2M communication investigating distinctive futures, challenges, and solutions, and reference [21] is a paper dividing the M2M communications into capillary M2M and cellular M2M technology, studying the challenges and opportunities. Thus, when aggregating the research on IoT as studied in references [11,12,13], and M2M communication as also studied in references [14,15,16,17,18,19,20,21], we can compare those two technologies to see which is more suitable and for what it is needed (Figure 5).

The main difference between M2M communication and IoT is that M2M uses a point-to-point communication. IoT device communication is based on a cloud wireless-network connectivity allowing for a large scale of integration. Scalability is a key difference. The IoT communication is highly scalable compared to M2M since the devices can be added to an existing network with minimum hassle. Whenever we refer to IoT communication, we always refer to wireless device communication with a comparison to M2M communication which includes both wired and wireless communication. Using wireless communication has its advantages and disadvantages, including less technology maintenance such as ethernet connectivity, but it also has less reliability. So, the use of M2M communication is best for applications that require point-to-point communication. Scalability is not a concern, and the application should be operational regardless of the existence of a Wireless Fidelity (WiFi) connectivity and needs to be executed quickly and reliably. On the other hand, for IoT communication, scalability is a concern, fast WiFi connectivity is needed, real-time synchronization is also needed, and multiple-standard compatibility is a must, as studied in reference [4]. The limited storage of smart nodes or smart sensors is another disadvantage of M2M communication. Smart devices, in order to save power consumption, have to disconnect from their wireless connectivity. The above may lead to important data loss. This is an issue that could be solved by processing and storing the important data, but this might have higher power consumption.

Talking about M2M communication, as for future research, there are many challenges that need to be analyzed and solved. The M2M communication is still at an early development stage. Such challenges are, first of all, scalability. Network congestion, system overload, and the limited particular bandwidth are an issue in the number of devices that will need to be served and perform network authentication. Also, M2M communication will produce more traffic of uplink (UL) connectivity than downlink (DL) connectivity. The use of lightweight symmetric cryptography seems to be quite interesting to be used instead of asymmetric in terms of computing and energy consumption. Real-time communications, such as eHealth or eCall applications, should be handled with a high Quality of Service (QoS).

## 3. The 5G Network

### The 5G Network Evolution

Based on our thorough research on references [22,23,24,25,26], the 5G mobile network technology will deliver a massive system capacity, extremely high data rates, very low latency, and ultra-high reliability and availability. New applications like remotely controlled robots and self-driven cars will impact industries and consumers. New radio technologies have been developed and designed like Long Range (LoRA) and SigFox used for MTC, as mentioned in references [14,15,16,17,18,19,20,21], with very limited demands on throughput, reliability, and QoS. LoRA as reviewed in references [27,28,29,30], provides a long-range with low-power secure data transmission, operating within the frequency bandwidth range of 915 to 928 MHz. SigFox based on the research in references [31,32,33,34,35] is a low-power network operating within the frequency range of 915 MHz to 928 MHz, providing high capacity with low-power requirements as mentioned in references [36,37,38].

The architecture of the 5G network will be based on the demands on human-to-human and M2M types of communication tuned based on user requirements. 5G is the network prepared to bring challenges such as connectivity to an enormous number of connected devices, most of them being supported by virtualized networks minimizing the equipment cost and of course reducing any implementation complexity. Virtualization as a technology can boost the backhaul and reduce the processing load of the end devices, fulfilling requirements such as energy constrain and size reduction of the IoT devices. In 5G, the main challenge we face is the strong backhaul, which is provided by virtualization. The 5G network is a unified network appearing to the end user as one system. QoS guarantees reliability with a packet-loss rate of 99.99% for this network in terms of connectivity for a variety of M2M services. To be more specific, the 5G network, using cloud technology and virtualization, is meant to be the one that will meet all of the reliability and latency requirements in terms of M2M communication, allowing for maximum end-to-end connectivity latency, including retransmissions less than 5 ms.

O-RAN empowers the 5G network, providing flexibility and freedom to network operators without slowing down any operations, but apart from that, the main weakness is the incapability of its control application to address latency less than 10 ms of time frame. O-RAN in 5G reduces network Operating Expenses or Expenditure (OPEX) and Capital Expenditures (CAPEX), and this is achieved by using open interfaces, allowing for the increase of market competition. Cloud-native O-RAN also enables open-source software to be applied as a result of fast innovation, improving network efficiency and performance. O-RAN will allow for QoE optimization, massive MIMO optimization, and traffic steering.

Existing work on 5G includes reference [22], which is a comprehensive review of the M2M communication, investigating distinctive futures, challenges, and solutions. Ref. [23] is a comprehensive study focusing on the M2M technologies, futures, challenges, and opportunities, investigating the multiplicity of the M2M communication. An overview paper is a reference [24] of the current standardization practices, listing solutions for the 5G spectrum requirements. Ref. [25] is an overview of the challenges and key features of the 5G mobile networks. Ref. [26] is a comprehensive overview of the long-term future of 5G mobile communications. Ref. [27] provides a basic understanding of the LoRa and Long-Range Wide-Area Network (LoRaWAN) radio communication technology. Ref. [28] is a Wikipedia article related to LoRa and LoRaWAN radio communication technology. Ref. [29] is a .pdf file that provides basic information related to LoRa as modulation. Ref. [30] is a webpage related to the spreading factor as a performance key characteristic of LoRaWAN. Information about the right balance between long-range communication and battery performance is provided. Ref. [31] is a review of applications, technologies, and challenges met in the 5G network, discussing the 5G network architecture, adaptive service architecture, data privacy, synchronization, and reliability. Reference [32] is an in-depth analysis of fog-computing architecture and technology as a proposed solution for latency, bandwidth, and power issues. Reference [33] is a complete overview of the design, deployment, and performance of the LTE, LTE-A, IP Multimedia Subsystem (IMS), voice-over LTE (VoLTE), IoT, and 5G networks. As studied in reference [34] is a book that reviews Artificial Intelligence-based (AI-based) applications, aspects, and models. Reference [35], which is a book that focuses on energy efficiency, security, performance, and interference in different applications such as smart cities, healthcare, and transportation systems. Reference [36] is a survey paper that provides information on challenges and issues related to M2M communication over 3GPP, LTE, and LTE-A. Reference [37] is a paper focusing on the coexistence of waveforms within the 5G network with each service, and which one is best for use. Reference [38] is a study related to the technology elements for efficient small-sized drone detection with the use of the 5G millimeter-wave cellular network. Reference [39] is a guide on the aspect of network slicing of the 5G network infrastructure. Reference [40] is a study of solving issues related to network slicing and developing an efficient framework for 5G wireless and core networks. Reference [41] is a book that provides a comprehensive reference for all of the 5G network layers, focusing on fundamental issues and challenges of major areas such as capacity and spectral efficiency on both radio and core sites. Reference [42] is a review of the functionality of Release 16, but it also contains a lot of information on the coming Release 17 of the 5G network. Reference [43] is a technical coverage of 5G applications and services, as well as information related to network reliability and security. Reference [44] is a 3GPP standardization informational site related to the CUPS. Reference [45] includes detailed information of transportation, services, and network architecture on RAN, and reference [46] is a well-written book discussing the challenges of massive IoT technology within the 5G network, intelligent transportation systems, green communication, and multi energy systems in smart cities. Reference [47] is a description of the local and metropolitan area networks, including WiFi, 4G compared to LTE, and 5G and its expectations.

The 5G mobile network has the full support of IPv6 connectivity. More specifically, when the UE requests an Internet IPv4 address in order to access an IPv4 service, the Packet Data Network Gateway (PGW), which is a part of the core, will allocate an IPv6 address. This IPv6 address will be used towards all the external interfaces, so the session in the PGW will be handled as an IPv6 session. Then, the UE will receive a dummy IPv4 address, and the uplink payload headers will be translated from an IPv4 to an IPv6, and the downlink payload IP headers will be translated from IPv6 to an IPv4. A benefit of all the above is that we maintain the legacy IPv4 business, with the UEs working in an IPv6 network achieving IPv4 address shortage mitigation, which reduces the complexity of IPv4-to-IPv6 migration.

The 5G network has the full support of network slicing as studied in references [39,40,41,42], which means that there can be a deployment of multiple logical networks (what we call slices) on a common physical infrastructure. These slices are isolated from each other on the control plane part and the user plane part (CUPS functionality), as well as the management plane. This allows for the slices to be optimized individually. This optimization of the slices can be in terms of performance, functionality, and geographical deployment.

Studying the separation of the CUPS for the network functions on references [43,44], provides us the possibility of investigating the independent scalability of the control, the user plane, and also the additional deployment flexibility. A user plane function supporting a low-delay application can be deployed closer to the access, while the control plane can be placed at a more centralized location.

**Figure 6:**
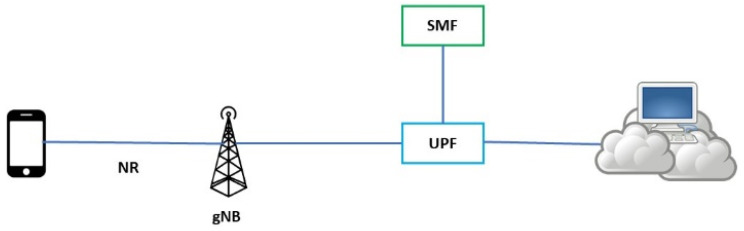
5G end-to-end communication, including CUPS functionality.

Discussing RAN architecture in 5G in references [41,42,43,44,45,46] (Figure 6), we should consider security.

The transport network must be able to handle the advanced security algorithms without introducing a delay in the transmission. Also, compliance with more stringent regulatory and industrial standards with respect especially to security is required, as well as compliance with an operator or service provider’s security policies. To protect the new and, of course, the existing RAN interface, IPsec is recommended. IPsec is used to secure the traffic from the baseband to the core network, but there is more IPsec directly between Evolved Node B (eNB) and G Node B (gNB). IPsec ensures secure communication over untrusted transport networks with “confidentiality” in order to keep data secure and hidden, “integrity” ensures no one has modified the data, and authentication authenticates the originating and receiving devices as trusted sources and anti-replay protection to avoid replay from previous recording. There will also be more decentralization of the security gateways (SeGW), as mentioned in reference [47], which are positioned closer to the radio sites in order to reduce latency.

Also, network firewalls will be required to cope with the increasing data volumes, which means there will be a need for hardware optimization. The eNB and gNB rely on asymmetric cryptography and digital signatures in order to authenticate the communicating peers and also validate signed files. Certificates and trust relationships are required in order to ensure the correctness and validity of the used keys. These node specific and trusted certificates are vendor credentials used to authenticate the network-access, node credentials are used for operation and maintenance (O&M) communication, chain certificates for node credentials, and trusted certificates are used to validate peer certificates.

The 5G technology has many advantages, but there are also many disadvantages. Among the advantages, we include the low latency, with round-trip data transmission taking less than five milliseconds and increased bandwidth, allowing for greater optimization of the network traffic. In terms of capacity, there is an enhancement, resulting in a capacity up to 1000 times greater than the 4G network, preparing the ground for 5G connectivity. In terms of security, we can have strong encryption, which is 10 times more secure than what the 4G network can offer. Further, 5G also allows for a great number of connected devices, allowing millions of connections per square kilometer.

It is quite interesting to examine the disadvantages of 5G networking and the need to find innovative solutions in order to overcome the issues. One of the biggest challenges of 5G is that there will be a need for many cellular base stations to be deployed on small distances due to the radio spectrum used. Radio waves need clear paths in order to reach the destinations. Mountains, trees, buildings, or even human bodies can block the 5G signals, especially in urban areas. Even the air can absorb a part of the signal waves. The above, of course, leads to high deployment costs, which is one of the biggest business disadvantages that future researchers should consider. Existing incompatible hardware is also another issue in processing 5G connectivity. In terms of security, we can say that this is one of the main research gaps and open challenges to be covered and solved. Any new technology always brings new security concerns. Any organization using the 5G technology should consider any network cyberattacks. Most of the existing protocols work for a particular, and not multiple, attack. Therefore, there is a need for designing protocols that will protect the network from multiple attacks. Additionally, IoT applications using the 5G network will require security protocols to fulfil their communication needs and data protection. New, efficient protocols are needed.

## 4. The 6G Network

### The 6G and Its Challenges

Our survey in the new era of 6G technologies and architecture has proven to us that we are currently moving from the concept of a “connection of things” to the “connection of intelligence” as reviewed in references [48,49,50,51,52,53,54,55]. With the rapid development of wireless communication, 5G will not be enough to meet its growing demand. It is hard to predict how exactly 6G network can meet this growing demand, although it is expected to be in use around the year of 2030 with the initial deployment being available only to business and high-performance applications.

**Figure 7:**
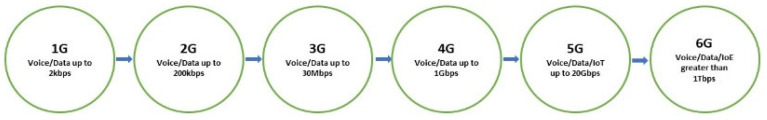
The mobile network evolution from 1st to the 6th generation.

Related existing work on 6G is the reference [48], which is a review of the 6G network with its key technologies including virtual, augmented, and mixed reality. Reference [49] does explores challenges and ways of improving future applications security within the NextGen networks and protection of user data from illegal access. Reference [50] is a survey paper related to the recent activities and trends for the 6G networks performing 5G and 6G using case analysis and future connectivity solutions. Reference [51] analyzes the development of 6G antenna technology covering also challenges of massive 6G IoT networks. Reference [52] is a book focusing on the wireless and optical domains of the 6G networking and discussing the interoperability and benefits of such technologies. In reference [53], we see an overview of the network evolution from our current 5G to 6G, outlining research directions and exploring the expected application and requirements. Reference [54] is a book focusing on machine learning, SDN, 5G and 6G networking, “cloud computing”, and “deep-learning” solutions. Reference [55] is exploring the past, present, and future wireless networks, as well as future 6G network application potentials, requirements, challenges, and opportunities. Reference [56] is a paper reviewing previous and current Cyber–Physical System (CPS) architecture, proposing Service-Oriented Architecture (SOA) as a general flexible CPS architecture. Reference [57] is a joint Press Release for announcing the use of a silica glass antenna in order to transmit and receive 28 GHz 5G radio on vehicles and trains. Reference [58] is a press release on a prototype using transparent dynamic metasurfaces to manipulate 28 GHz 5G radio waves. In reference [59], we see a survey paper of Simultaneous Localization and Mapping (SLAM) for autonomous driving, providing an overview of the experiments carried out until now while discussing challenges and future orientations. Reference [60] is an article reviewing from 1G up to 5G services, and then highlighting what 6G services will look like based on what users really want. Reference [61] is a paper presenting an efficient and low-complexity deep-learning-based detector for Free Space Optical (FSO) communication systems. Reference [62] is an article performing simulation results, confirming that Momentum-Federated Learning (MFL) has significant convergence improvement over federated learning (FL). In reference [63], we see a paper presenting the way of wireless charging technique of mobile applications over the ether with the use of Distributed Laser Charging (DLC) and in reference [64] we do review a paper presenting the Low-power Wakeup Radio (WUR) for applications improving significantly the performance of wireless sensors. The 6G network will be there to support all this rapid growth and process large amounts of data (Figure 7).

Discussing differences between 5G and 6G, 6G will be a network that will have the needed capacity and transmission rates. IoE comes fully into the picture, which is the acceleration of IoT but with more intelligence. Based in reference [56], IoE can actually connect the real world to the virtual in order to create a better human world that knows our desires and preferences as human beings and is able to perform tasks based on that automatically.

The 6G network will be the network to use higher frequencies compared to 5G, providing a higher capacity and much lower latency, which supports up to one millisecond latency communication. The above will solve any communication issues that currently exist on 5G networks that are between “Humans and Things”. Such communication will be the wearable and micro-devices, which are mounted into the human’s body, five-sense communications, and 8K holograms, and there are also expectations to support extreme massive connectivity and the transmission of data, which supports 10-million devices per square km. All of the above, will dramatically eliminate any cultural and social differences between urban and rural areas. From an architectural point of view, the main difference between 5G and 6G networks is that the 5G network relies on using small base stations equipped with mm wave cells, while 6G uses cell-free smart surfaces using high frequencies.

In terms of using the O-RAN on the 6G network, O-RAN enables AI service control for the RAN Intelligent Controllers (RICs) supporting AI service chaining per device. In simple words, 6G will transform the communication of IoT into the connection of intelligence using ML and deep learning (DL), which supports massive interconnectivity, flexibility, and energy efficiency. It is also a challenge to fully adopt O-RAN in the 6G network since this requires the merging of 3GPP and O-RAN standardization specs into one. Security is an issue that is considered in 6G since the amount of data and traffic will be huge. Privacy, confidentiality, and trust based on our survey are topics in 6G that will be a real challenge.

Furthermore, 6G will be the network to interconnect all things. It will integrate the ground communication with the satellite communications and marine communications. If we consider that more than 70% of earth is covered by water, the increase of marine applications will require full network coverage over and under the water as well. Also, 3D communication will allow for the possibility of integrating ground and airborne networks. Although full coverage across the whole planet with enough capacity is quite far from reality and very costly. There are also many research papers regarding the network topology of 6G, with some of those suggesting the use of glass antennas and reflectors, as studied in references [57,58]. Extensive solutions will also be needed since there will be a need to support connectivity for flying drones, space stations, and ships since the 5G network is not able to support that.

**Figure 8:**
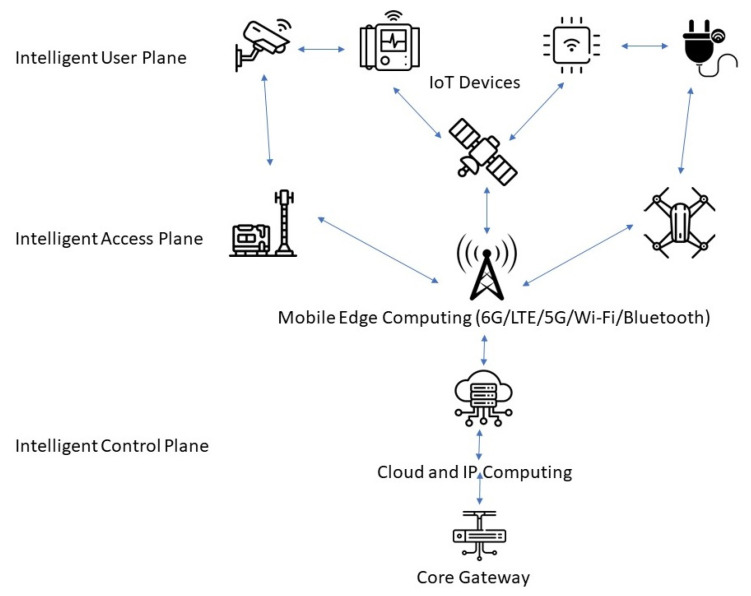
The 6G network architecture.

Figure 8 shows a very basic diagram of the 6G network based on a series of assumptions. All areas are based on intelligence, the Intelligent User Plane area, the Intelligent Access Plane area, and Intelligent Control Plane areas.

The Intelligent User Plane is the one that can collect all of the information from the IoT devices sensors and then pass all of this information to the Intelligent Access area supported by Mobile Edge Computing connectivity, and then that will pass all the information the core gateway in order to handle them.

To meet all of the coming challenges, the 6G mobile network will provide all of the technical standards and techniques required. The 6G network is actually what follows the 5G network and tries to make it more complete. One of these challenges is the possibility to use a terabit wireless network to transfer data. In order to achieve this speed, we will need to transmit signals above 1 terahertz. For that, we need a new chip design, computing architecture, and energy sources. This will have a huge impact on IoT applications such as medical imaging, automated driving, smart home, etc. Car self-driving will be able to sense the environment and, based on the information received, be able to update all of the information in real time such as map info, which will allow cars to keep and update location info in extreme network speeds. This technology is called Simultaneous Localisation and Mapping (SLAM) and allows for the building of a map of a vehicle’s surroundings environment info using cameras and sensors based on reference [59].

The 6G network is expected to provide great capacity compared to 5G, with ultra high-speed data connectivity and ultra-low latency supporting new applications such as surreal virtual reality, disaster prediction, etc. To be more specific here, we are talking about data rates of up to 1000 times faster than what we can achieve with 5G. Talking about capacity, 6G compared to 5G will make it possible to connect up to a trillion objects, compared to the billion that we currently have in 5G as reviewed on reference [60]. In terms of latency, 6G will provide improvement compared to 5G, which is 1ms smaller than that (about <1 ms).

The 6G network will support the connectivity demand of robotics and autonomous drone system applications that can be handled with the 5G network, although 6G is the most optimal because of its low-latency specs. Further, 6G will be a network with human intelligence and will provide ways to communicate with smart terminals. It will support mobile internet, IoT, holographic, and precision communications, which requires a low latency and high throughput of data.

ML is a key topic that deals with smart applications in the 6G network. ML’s main weakness is the issue with privacy and high overload of centralized servers and also requires high-power consumption in order for large datasets to be processed, as mentioned in reference [61]. Distributed machine learning comes as a solution to the above issue by enabling parallel computation. This model is based on the division of data between the number of nodes, with all of them having the same machine-learning model.

According to reference [62], in 6G network deployment, we have to take into consideration the data-transmission issues for long distances such as a high-path loss since THz waves are the ones that can provide high data rates. There is a need for a new transceiver architecture design in order to be able to operate in such a high frequency. The transceiver needs to be able to operate and communicate in high frequencies and make sure that very wide bandwidth is fully utilized.

Device energy is a huge challenge in the 6G network, even more than in 5G. According to reference [63], there has been a lot of investigations ongoing on that topic, and there is a possibility of achieving the charging of the mobile devices to be performed using the radio waves or laser beams together with energy harvesting, solar panels, or even energy achieved from walking activity to support power requirements for the IoT devices. The energy harvesting of circuits of the devices can be self-powered solving issues, such as long-lasting energy support, which is one of the most critical issues we are facing in the IoT devices. Another investigation ongoing on energy saving is the Wakeup Radio (WUR) as seen on reference [64], which is mentioned that the devices are put in a sleep-mode until the radio sends a signal to the device to wake up. During this sleep-mode time, the device will be consuming no energy or a very small amount of it.

It is good to mention any affects that THz waves could have on human health and safety. There are a lot of on-going studies in order to investigate any issues we might face on that level.

As 6G network benefits, we can say that it is the network that is combining and corelating different technologies allowing high speed with low-latency communication compared to 5G. It will enable new application capabilities and also extend existing ones, with ultimate security and privacy providing safeguards against treats. It is the network that focuses on power consumption optimization and improves cellular penetration reducing interference between devices. There will be unmatched speed achieving more than 1 terabit per second (Tbps), and technologies such as machine learning (ML) and Artificial Intelligence (AI) will fully come into the picture, reaching superior efficiency. In terms of disadvantages and research gaps for further investigation, we can say that one of the main ones are health-risk-related exposures on high-radio frequencies. The energy consumption is, of course, another risk, since there will be a speed and capacity increase compared to 5G, with the potential risk of data privacy and loss being one of the main topics. Hardware infrastructure cost is also another important area for future research since there will be requirements for significant development, and new protocols will be needed in order to support all the new functionality.

## 5. Discussion

By this paper, we can see that 5G and 6G technologies are the technologies expected to be used for the connectivity of Internet of Things (IoT) and Internet of Everything (IoE) changing the way people interact with each other. Those technologies will dramatically change our lives since they will be used for indoor- and outdoor-device connectivity. Machine-to-machine (M2M) communication is analyzed as playing an important role within the 5G network since it is the type of communication that will be controlling devices such as vehicles that will require real time and immediate responses.

With this paper, we can understand that the 6G network will offer a completely new communication experience compared to what 5G can offer to us interconnecting all things, being the connection of intelligence bringing in full functionality technologies such as Artificial Intelligence (AI) and machine learning (ML), providing great capacity.

We can see that as we move towards 6G with all its supported applications, the main issue is energy and more specific battery lifetime. Battery life is an issue that is improving, but it is not completely solved, and it is the major factor affecting the IoT devices.

Within this paper, we see that device energy is a huge challenge in 6G network, a critical topic for future work and investigation requiring new approaches such as energy harvesting with self-powered circuits.

## 6. Conclusions

Within this survey paper, we have explored a series of capabilities we actually have within the 5G network and series of challenges we will face with the use of the 6G network. Further, 5G plays a significant roll on the IoT device connectivity capacity in order to transmit all the needed extremely large amount of real-time data the sensors collect from substantial number of connected devices. The 5G network will fulfill the gap of the 4G network in order to handle all these information becoming the backbone for IoT connectivity for the currently needed capacity.

Motivated by the lack of information on 6G, the 6G network will be the one that will actually unlock full functionality of smart cities by enabling the Internet of Everything (IoE). The 6G network will be a major step forward.

The 6G network will actually enable services that we cannot think of right now. Artificial Intelligence (AI) and machine learning (ML) will support new capabilities and improve performance issues that we currently have in the 5G network. Terahertz communication will be used as a communication band within the 6G network.

Ultra-long battery lifetime, energy harvesting, and wireless charging will be provided. The extensive use of machine learning will also be applied.

## Data Availability

Data are contained within the article.

## Abbreviations

Acronym	Definition
IoT	Internet of Things
IoE	Internet of Everything
CUPS	Control Plane and User Plane Separation
LPWA	Low Power Wide Area
QoS	Quality of Service
NR	New Radio
MIMO	Multiple input, Multiple output
HD	High Definition
M2M	Machine to Machine
UP	User Plane
PCRF	Policy and Charging Rules Function
OCS	Online Charging System
SDN	Software Defined Network
VNF	Virtual Network Function
OSI	Open Systems Interconnection
PSM	Power Saving Mode
eDRX	Extended Discontinuous Reception
PDN	Packet Data Network
UE	User Equipment
MME	Mobility Management Entity
HLC	High Latency Communication
MT	Mobile Terminated
AES	Advanced Encryption Standard
ECC	Elliptic Curve Cryptography
LoRA	Long Range
MTC	Machine-Type Communication
PGW	PDN Gateway
IPv4	Internet Protocol 4
IPv6	Internet Protocol 6
RAN	Radio Access Network
SeGW	Security Gateways
O&M	Operation and Maintenance
SLAM	Simultaneous Localization and Mapping
ML	Machine Learning
WUR	Wakeup Radio
AI	Artificial Intelligence
